# Supplementary material for: Cross-reactive neutralizing human survivor monoclonal antibody BDBV223 targets the ebolavirus stalk
Source: Nat Commun. 2019 Apr 17;10:1788. doi: 10.1038/s41467-019-09732-7 (PMC6470140; doi:10.1038/s41467-019-09732-7)
Supplement: Supplementary file 1 — Supplementary Information [file 41467_2019_9732_MOESM1_ESM.pdf]

Supplementary Information

Cross-reactive neutralizing human survivor monoclonal antibody BDBV223 targets the ebolavirus stalk

King et al.

Supplemental Table 1

BDBV223-peptide	
Resolution range (Å)	38.55 - 3.68 (3.82 - 3.68)
Space Group	$P\ 1\ 2_1\ 1$
Unit Cell (a,b,c in Å; α,β,γ in °)	a = 51.45, b = 191.58, c = 63.36<br>α = 90, β = 109.98, γ =120
Total reflections	41,422 (3,986)
Unique reflections	12,104 (1,176)
Multiplicity	3.4 (3.4)
Completeness (%)	97.1 (96.4)
I/σ(I)	3.7 (1.7)
R_{merge}	0.31 (0.68)
R_{pim}	0.19 (0.43)
$CC_{1/2}$	0.95 (0.79)
Wilson B ($Å^2$)	54
Reflections used in Refinement	12104 (1176)
R_{work} (%)	22.8 (29.0)
R_{free} (%)	25.4 (38.3)
Number of non-hydrogen atoms	6,913
Macromolecule atoms	6,913
Water	0
Protein residues	900
RMS (bonds) (Å)	0.00
RMS (angles) (°)	0.68
Ramachandran favored (%)	98
Ramachandran outliers (%)	0.0
Clashscore	4.1
Average B-factor ($Å^2$)	55

Supplemental Table 2

BDBV223 apo-Fab	
Resolution range (Å)	45.63 - 2.03 (2.10 - 2.03)
Space Group	$P\ 2_1\ 2_1\ 2_1$
Unit Cell (a,b,c in Å; α,β,γ in °)	a = 64.97, b = 75.68, c = 114.41<br>α = 90, β = 90, γ = 90
Total reflections	236,416
Unique reflections	37,109 (3,647)
Multiplicity	6.1 (5.7)
Completeness (%)	99.82 (99.70)
R_{pim}	0.04 (0.26)
$CC_{1/2}$	0.95 (0.85)
Wilson B (Å^2)	50
Reflections used in Refinement	37109 (3645)
R_{work} (%)	23.5 (28.2)
R_{free} (%)	25.0 (29.3)
Number of non-hydrogen atoms	3324
Macromolecule atoms	3324
Water	0
Protein residues	422
RMS (bonds) (Å)	0.01
RMS (angles) (°)	1.12
Ramachandran favored (%)	95
Ramachandran outliers (%)	0
Clashscore	5
Average B-factor (Å^2)	60

Supplemental Table 3. Alternate antibody numbering

Heavy Chain		
Residue	Sequence	IMGT
CDR H1		
G	26	27
G	27	28
S	28	29
F	29	30
T	30	35
T	31	36
T	32	37
Y	33	38
W	34	39
N	35	40
CDR H2		
E	50	55
V	51	56
N	52	57
Y	53	58
S	54	59
G	55	63
N	56	64
A	57	65
N	58	66
Y	59	67
N	60	68
P	61	69
S	62	70
L	63	71
K	64	72
G	65	74
CDR H3		
T	96	105
S	97	106
R	98	107
I	99	108
R	100	109
S	101	110
H	102	111
I	103	111A
A	104	112B
Y	105	112A
S	106	112
W	107	113
K	108	114
G	109	115
D	110	116
V	111	117

Light Chain		
Residue	Sequence	IMGT
CDR L1		
R	24	24
A	25	25
S	26	26
Q	27	27
S	28	28
V	29	29
P	30	30
R	31	36
N	32	37
Y	33	38
I	34	39
G	35	40
CDR L2		
G	51	56
A	52	57
S	53	65
S	54	66
R	55	67
A	56	68
A	57	69
CDR L3		
H	90	105
Q	91	106
Y	92	107
D	93	108
R	94	109
L	95	114
P	96	115
Y	97	116
T	98	117

Figure S1.

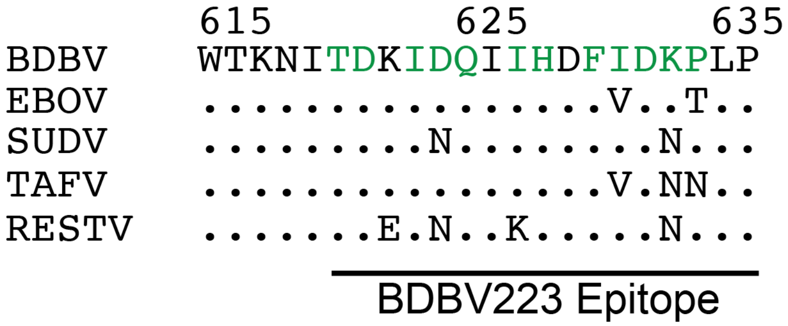

Figure S1. Sequence Alignment. Alignment of GP2 sequences of different ebolaviruses in the BDBV223 stalk epitope. Residues written in green are observed to interact with BDBV223 in the crystal structure.

Figure S2.

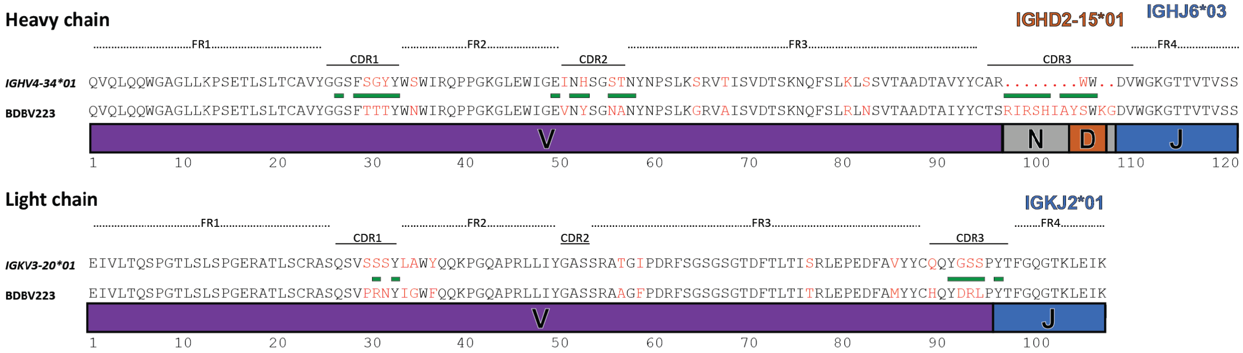

Figure S2. Somatic hypermutation of BDBV223. Alignment of the mature BDBV223 antibody heavy and light chains and to its IGHV-34*01 heavy chain and IGKV3-20*01 light chain human germline sequences. Green bars indicate peptide-interacting residues. Residues numbered according to primary sequence numbering (see **Table S3** for IMGT numbering). Abbreviations: V = Variable, N = Non-templated, D = Diversity, and J = Joining segments.

Figure S3.

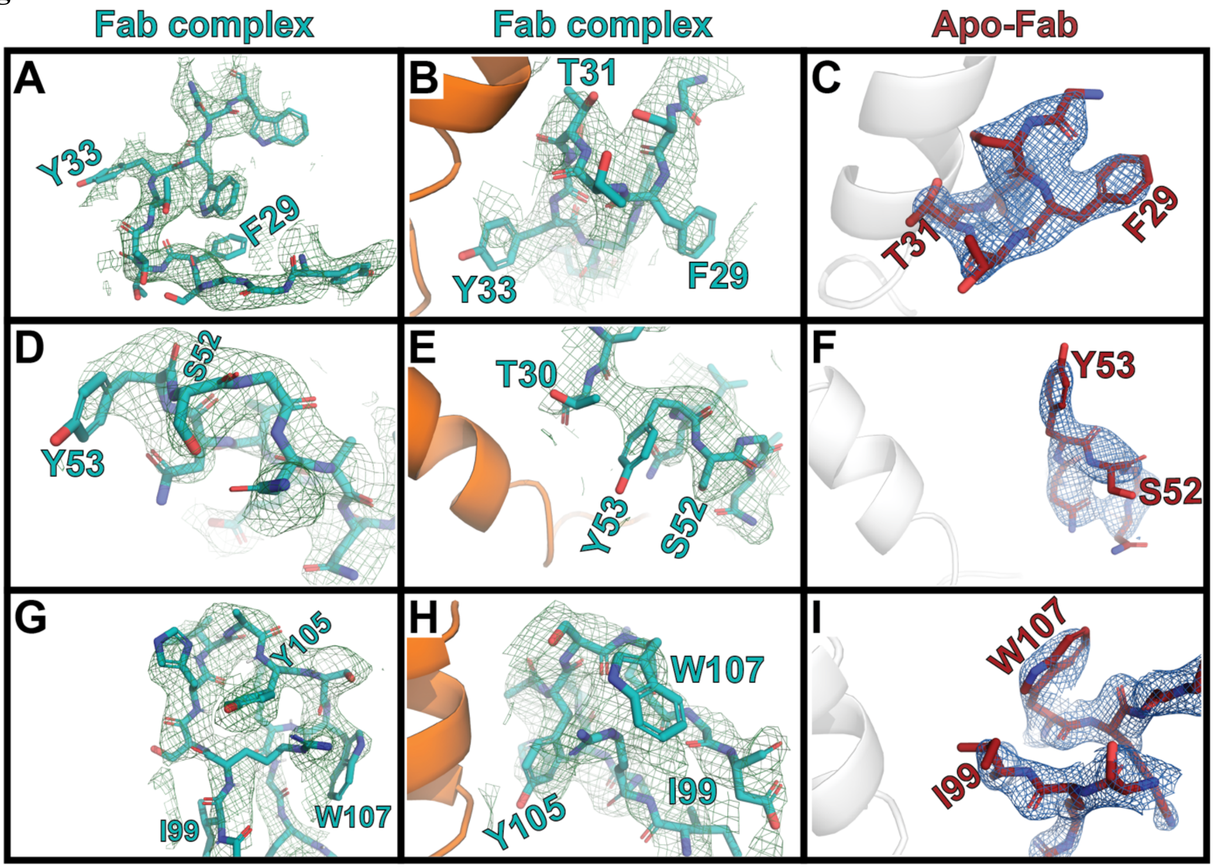

Figure S3: Electron density maps of heavy chain CDRs. Simulated annealing composite omit maps are illustrated for both the Fab-peptide complex and Apo-fab structures at 1.0 sigma. Panels **A**, **D**, and **G** illustrate each entire CDR in the Fab-complex structure. Panels **B, E** and **H** illustrate interactions of each CDR (teal) with the bound BDBV GP peptide (orange). Panels **C, F** and **I** illustrate the CDRs in the unbound (apo) Fab structure. Relative position of the BDBV GP peptide (white) is modeled to better illustrate differences in conformation and position between free (red) and complexed antibody (teal, panels **B, E** and **H**). Views approximate the views shown in Figure 2.

Figure S4.

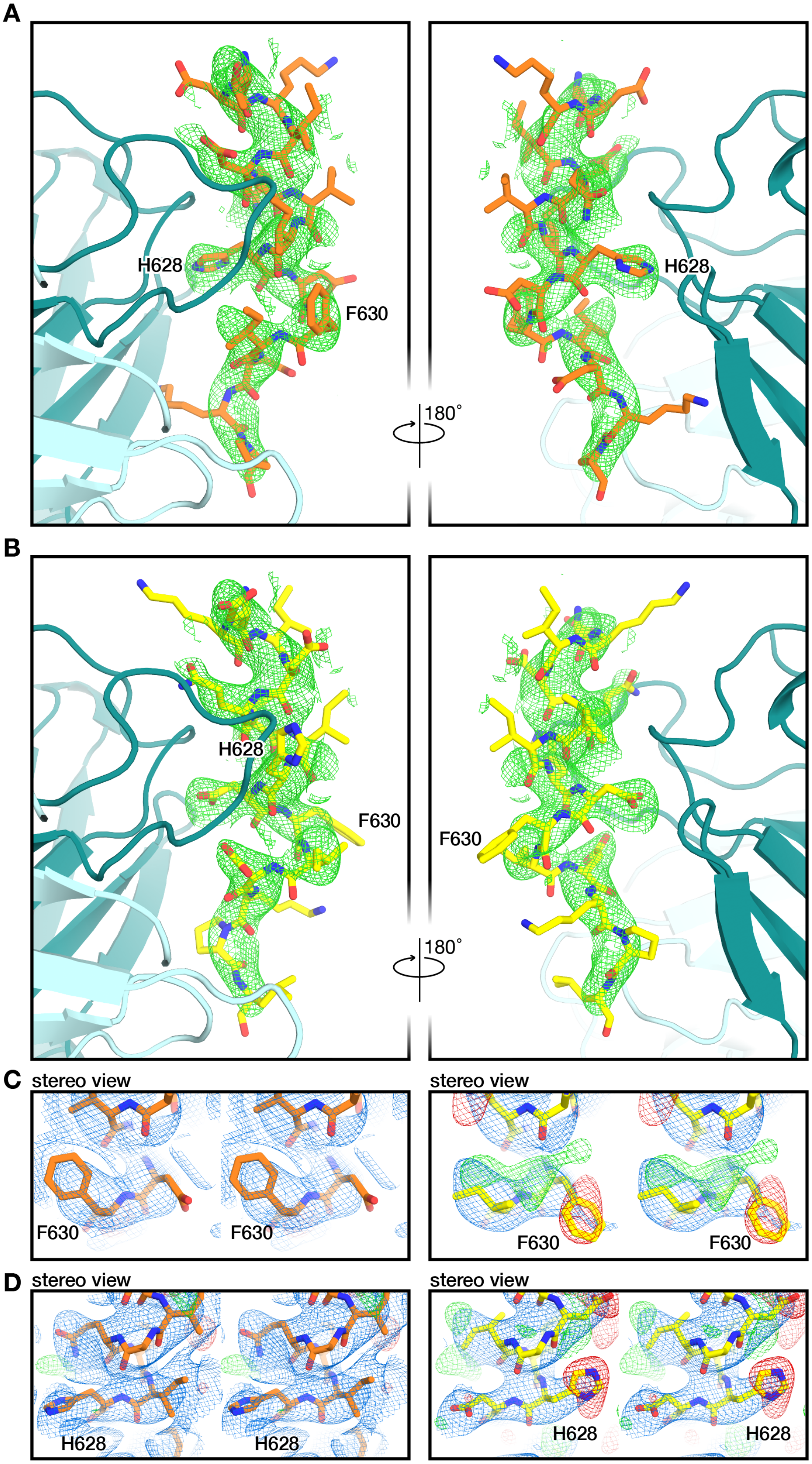
A
H628
F630
H628
180˚
B
H628
F630
F630
180˚
C
stereo view
F630
F630
stereo view
F630
F630
D
stereo view
H628
H628
stereo view
H628
H628

Figure S4: Electron density maps of GP peptide. (A) View of peptide modeled as in PDB:6N7J showing residues 620-634 of the peptide (orange). Green mesh represents the Fo-Fc density from a simulated annealing composite omit map calculated for the structure with the peptide deleted. **(B)** The same map as generated in panel **A** but with the peptide (yellow) register shifted to display residues 621-635 (maps displayed in panels **A** and **B** are contoured at 3 sigma, only positive density is visible in these panels). F630 **(C)** and H628 **(D)** of the peptide can be seen for each register after a round of refinement. Blue mesh represents 2Fo-Fc density, and green/red mesh represents Fo-Fc density. The left panel shows the result with the orange peptide modeled into the density, and the right panel shows the result with the yellow peptide modeled in (blue contoured at 1 sigma, green/red contoured at ±3 sigma). These maps indicate a significantly stronger fit when the 620-634 (orange) model of the stalk peptide is used. 3-dimensional visualization can be seen in Supplemental Video 1.

Figure S5.

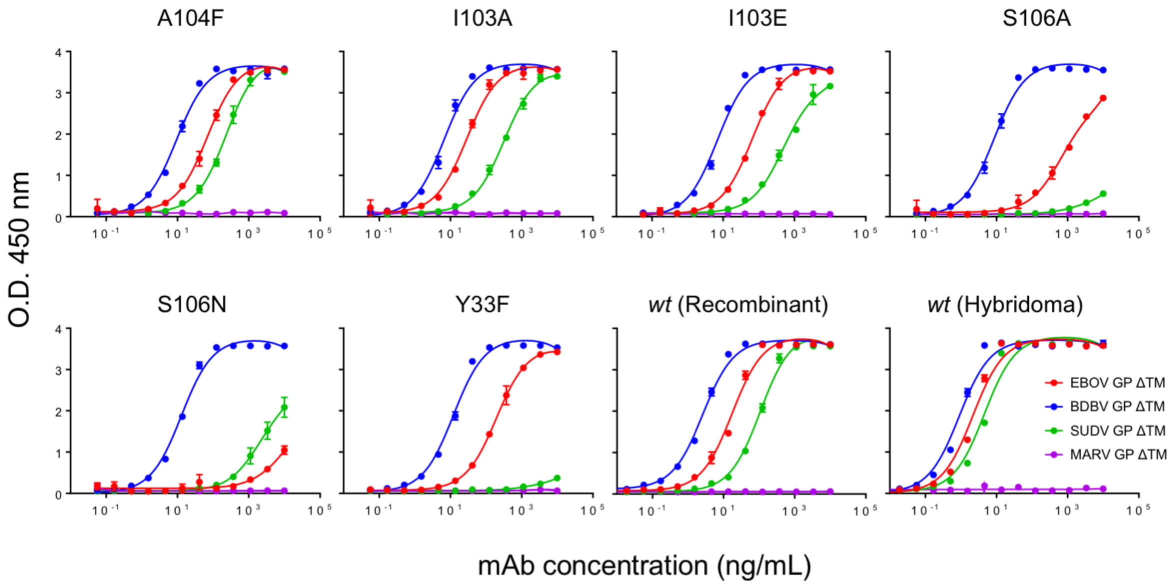

Figure S5. Reactivity of single amino-acid variants of mAb BDBV223 assessed by ELISA

Binding curves for recombinant wild type (*wt*), hybridoma *wt*, or recombinant single point mutant variants of BDBV223 antibody to recombinant filovirus GP ectodomains in ELISA. Means ± SD of triplicates are shown. R100A and R100W were performed and were unable to bind either EBOV, BDBV, or SUDV GP.

Figure S6.

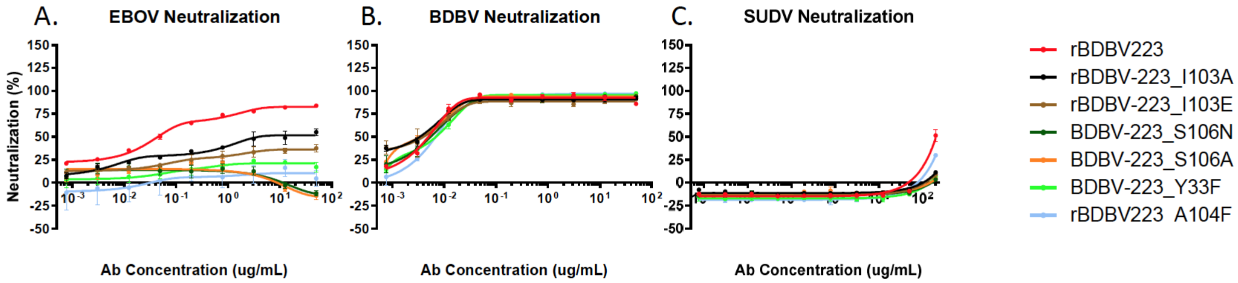

Figure S6. Neutralization of BDBV223 wild-type or variants against eGFP-expressing chimeric ebolaviruses. (A) Mutants showed varied neutralization capacity against EBOV while still maintaining full neutralization capacity of BDBV **(B)**. No mutants were able to achieve significant neutralization of SUDV **(C)**.

Figure S7.

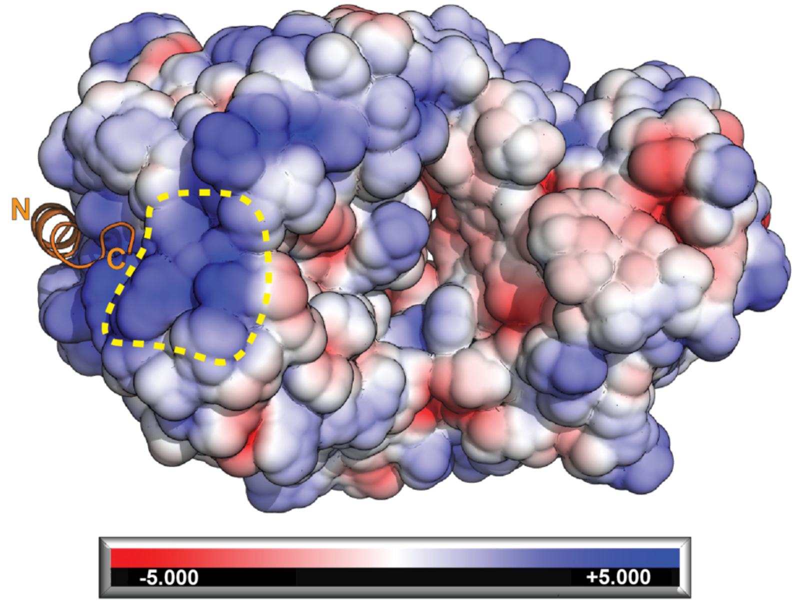

Figure S7. Electrostatic representation of membrane-oriented face of BDBV223. The underside of BDBV223 is represented in electrostatic surface representation, with red indicating a more acidic surface and blue indicating more basic (and white as neutral), and limits of +/- 5 $K_bTe_c^{-1}$. The paratope is at left and constant regions of the antibody at right. The bound GP stalk epitope (orange) is at far left. The yellow outlined region is a basic patch on the membrane-oriented surface of the light chain FR3 with basicity contributed by R94.

Figure S8.

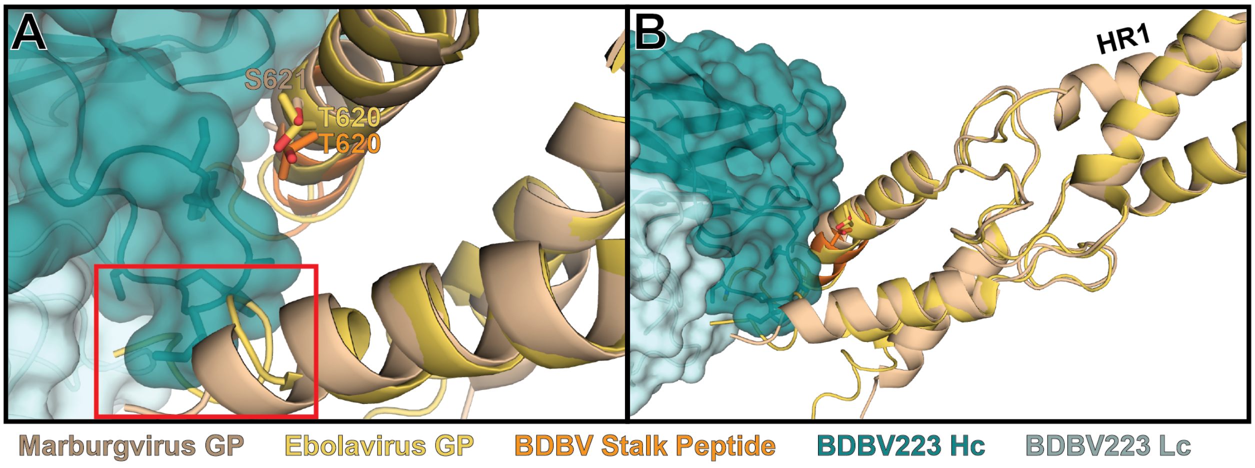

Figure S8. Marburgvirus clash alignment. Alignment was performed with Ravn virus GP (PDB: 6BP2), EBOV GP (PDB: 5JQ3), and the BDBV223-peptide complex structure (PDB: 6N7J) to evaluate whether the antibody would clash with the neighboring monomer in a structure without a fibritin foldon domain. Panel **A** demonstrates that some degree of steric clash would likely exist with both foldon-stabilized and native GP2 trimeric bundles. Panel **B** is shown to illustrate the strength of the GP alignments and only displays GP2 for clarity. Measured at the base of the helices, the Ravn GP stalks are separated by a total of 7.7 Å more than in EBOV GP.

Figure S9.

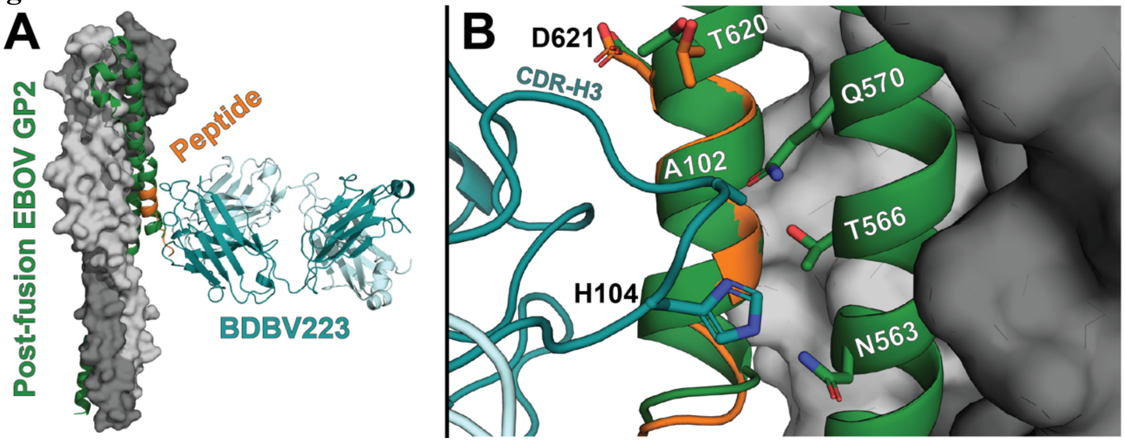

Figure S9. Superimposition of the BDBV223-peptide complex with post-fusion EBOV GP2. Alignment of the BDBV223-peptide complex (teal and orange) with the post-fusion structure of EBOV GP2 (green; PDB: 1EBO) indicate only two potential clashes (between A102 and H104 of BDBV223 and Q570, T566, and N563 of post-fusion GP2) .

Figure S10.

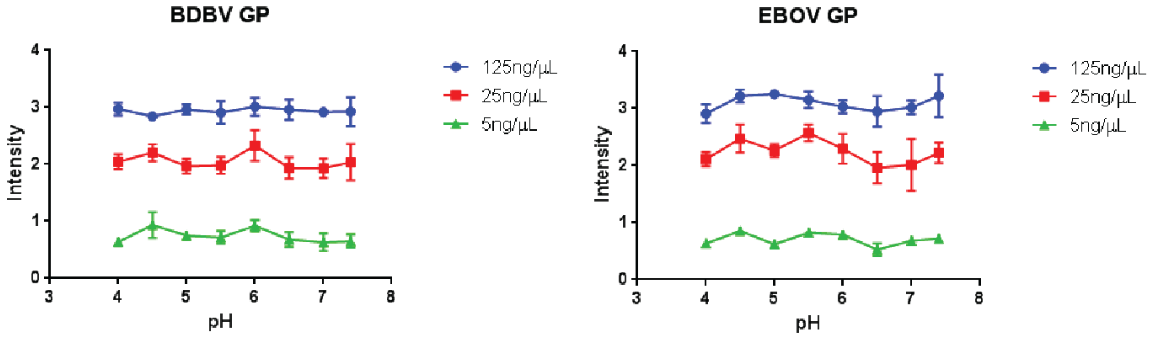

Figure S10. pH independence of BDBV223 binding. Binding of BDBV and EBOV GP in a pH gradient range of 4.0-7.4 by BDBV223 at concentrations of 5ng/mL, 25ng/mL, and 125ng/mL. The ELISAs show that pH does not affect the binding of BDBV223 to the GPs used. Optical density at 450nm, OD_{450}, was used to detect ELISA signal.
