# Supplementary material for: Cross-reactive neutralizing human survivor monoclonal antibody BDBV223 targets the ebolavirus stalk
Source: Nat Commun. 2019 Apr 17;10:1788. doi: 10.1038/s41467-019-09732-7 (PMC6470140; doi:10.1038/s41467-019-09732-7)
Supplement: Supplementary file 3 — Reporting Summary [file 41467_2019_9732_MOESM3_ESM.pdf]

nature research

Corresponding author(s): Liam King, Brandyn West, Crystal Moyer, Pavlo Gilchuk, Andrew Flyak, Philipp Ilinykh, Robin Bombardi, Sean Hui, Kai Huang, Alexander Bukreyev, James Crowe, Jr., and Erica Saphire

Last updated by author(s): Mar 11, 2019

# Reporting Summary

Nature Research wishes to improve the reproducibility of the work that we publish. This form provides structure for consistency and transparency in reporting. For further information on Nature Research policies, see Authors & Referees and the Editorial Policy Checklist.

## Statistics

For all statistical analyses, confirm that the following items are present in the figure legend, table legend, main text, or Methods section.

| n/a | Confirmed | |
|---|---|---|
| ☐ | ☒ | The exact sample size (*n*) for each experimental group/condition, given as a discrete number and unit of measurement |
| ☐ | ☒ | A statement on whether measurements were taken from distinct samples or whether the same sample was measured repeatedly |
| ☒ | ☐ | The statistical test(s) used AND whether they are one- or two-sided<br>*Only common tests should be described solely by name; describe more complex techniques in the Methods section.* |
| ☒ | ☐ | A description of all covariates tested |
| ☒ | ☐ | A description of any assumptions or corrections, such as tests of normality and adjustment for multiple comparisons |
| ☒ | ☐ | A full description of the statistical parameters including central tendency (e.g. means) or other basic estimates (e.g. regression coefficient) AND variation (e.g. standard deviation) or associated estimates of uncertainty (e.g. confidence intervals) |
| ☒ | ☐ | For null hypothesis testing, the test statistic (e.g. *F*, *t*, *r*) with confidence intervals, effect sizes, degrees of freedom and *P* value noted<br>*Give P values as exact values whenever suitable.* |
| ☒ | ☐ | For Bayesian analysis, information on the choice of priors and Markov chain Monte Carlo settings |
| ☒ | ☐ | For hierarchical and complex designs, identification of the appropriate level for tests and full reporting of outcomes |
| ☒ | ☐ | Estimates of effect sizes (e.g. Cohen's *d*, Pearson's *r*), indicating how they were calculated |

*Our web collection on statistics for biologists contains articles on many of the points above.*

## Software and code

Policy information about availability of computer code

| | |
|---|---|
| Data collection | Data was collected at beamlines APS 23-ID-D and SSRL 12-2 using software associated with the beamline |
| Data analysis | XDS, BUSTER, and Phenix were all used for data processing and structure refinement. GraphPad Prism was used as well for data analysis. |

For manuscripts utilizing custom algorithms or software that are central to the research but not yet described in published literature, software must be made available to editors/reviewers. We strongly encourage code deposition in a community repository (e.g. GitHub). See the Nature Research guidelines for submitting code & software for further information.

## Data

Policy information about availability of data

All manuscripts must include a data availability statement. This statement should provide the following information, where applicable:

- Accession codes, unique identifiers, or web links for publicly available datasets
- A list of figures that have associated raw data
- A description of any restrictions on data availability

Coordinates and structure factors have been deposited into the Protein Data Bank under accession numbers 6N7J and 6N7U for the complexed and apo BDBV223 structures, respectively. All other data is available from the authors upon reasonable request.

# Field-specific reporting

Please select the one below that is the best fit for your research. If you are not sure, read the appropriate sections before making your selection.

- [x] Life sciences
- [ ] Behavioural & social sciences
- [ ] Ecological, evolutionary & environmental sciences

For a reference copy of the document with all sections, see nature.com/documents/nr-reporting-summary-flat.pdf

# Life sciences study design

All studies must disclose on these points even when the disclosure is negative.

| | |
|---|---|
| Sample size | N/A |
| Data exclusions | No data were excluded. |
| Replication | All biochemical tests were performed in duplicate at a minimum, and all data observed were reported. |
| Randomization | N/A |
| Blinding | N/A |

# Reporting for specific materials, systems and methods

We require information from authors about some types of materials, experimental systems and methods used in many studies. Here, indicate whether each material, system or method listed is relevant to your study. If you are not sure if a list item applies to your research, read the appropriate section before selecting a response.

## Materials & experimental systems

| n/a | Involved in the study | |
|---|---|---|
| [ ] | [x] | Antibodies |
| [ ] | [x] | Eukaryotic cell lines |
| [x] | [ ] | Palaeontology |
| [x] | [ ] | Animals and other organisms |
| [x] | [ ] | Human research participants |
| [x] | [ ] | Clinical data |

## Methods

| n/a | Involved in the study | |
|---|---|---|
| [x] | [ ] | ChIP-seq |
| [x] | [ ] | Flow cytometry |
| [x] | [ ] | MRI-based neuroimaging |

## Antibodies

| | |
|---|---|
| Antibodies used | goat anti-human IgG HRP (Southern Biotech, CAT: 2040-05), BDBV223 (generated as part of previous study and provided by co-autho) |
| Validation | Goat anti-human IgG HRP was validated by manufacturer, reports available at (https://www.southernbiotech.com/?catno=2040-05&type=Polyclonal#)<br>BDBV223 is a human antibody developed as part of a previous study (Flyak, Ref #18) and has been validated structurally as part of this study. |

## Eukaryotic cell lines

Policy information about cell lines

| | |
|---|---|
| Cell line source(s) | HMMA2.5 myeloma cells. Mammalian 293F cells. Drosophila melanogaster S2 cells. |
| Authentication | These cells were not authenticated. |
| Mycoplasma contamination | These cells were not tested for mycoplasma contamination. |
| Commonly misidentified lines (See ICLAC register) | *Name any commonly misidentified cell lines used in the study and provide a rationale for their use.* |
