# Supplementary material for: Cross-reactive neutralizing human survivor monoclonal antibody BDBV223 targets the ebolavirus stalk
Source: Nat Commun. 2019 Apr 17;10:1788. doi: 10.1038/s41467-019-09732-7 (PMC6470140; doi:10.1038/s41467-019-09732-7)
Supplement: Supplementary file 4 — Description of Additional Supplementary Files [file 41467_2019_9732_MOESM4_ESM.pdf]

Description of Additional Supplementary Files

File Name: Supplementary Movie 1
Description: The camera rotates around the BDBV223-GP2 stalk peptide binding interface. The peptide is displayed in the two theoretical registers (620-634 colored in orange, and 621-635 colored in yellow). Electron density, shown in green, is positive density generated from a Simulated Annealing Composite Omit map displayed at +3 sigma. The published register (620-634 in orange) shows a stronger fit of the density than the 621-635 register.
